# Small bowel neuroendocrine tumor with scoliosis: A case report and literature review

**DOI:** 10.1097/MD.0000000000042395

**Published:** 2025-05-02

**Authors:** Cheng Deng, Jin Su

**Affiliations:** a Department of General Surgery, Medical Center of Digestive Disease, Zhuzhou Hospital Affiliated to Xiangya School of Medicine, Central South University, Zhuzhou, China; b Department of Teaching, Zhuzhou Hospital Affiliated to Xiangya School of Medicine, Central South University, Zhuzhou, China.

**Keywords:** case report, neuroendocrine tumor, scoliosis, small bowel, small bowel obstruction

## Abstract

**Rationale:**

Small bowel neuroendocrine tumors (SBNETs) often present with nonspecific clinical manifestations, which can complicate diagnosis and treatment when coexisting comorbidities in the perioperative period. This report discusses a rare case involving SBNETs associated with scoliosis, aiming to provide a comprehensive understanding of the epidemiological characteristics, clinicopathologic features, and treatment strategies related to SBNETs.

**Patient concerns:**

A 69-year-old male presented with a 10-month history of abdominal pain, nausea, vomiting, and weight loss. He had been admitted to the other medical institutions multiple times due to recurrent abdominal pain and was diagnosed with small bowel obstruction over the past 10 months. He had a history of scoliosis. Radiographic spine imaging revealed severe scoliosis. At the same time, contrast-enhanced abdominal CT scans indicated slight thickening and enhancement of the small intestinal wall and identified a mass in the mesentery. An enteroscopy did not reveal any significant abnormalities.

**Diagonses:**

Histopathological examination of the tumor specimens confirmed the diagnosis of a small bowel neuroendocrine tumor.

**Interventions:**

The case was reviewed in a multidisciplinary team discussion, which led to the decision for an exploratory laparotomy. During the surgical procedure, a segment of the small intestine and the associated regional mesenteric lymph nodes were successfully resected.

**Outcomes:**

The patient had an uneventful recovery after surgery, and a follow-up 6 months later showed no signs of recurrence.

**Lessons:**

Contrast-enhanced abdominal CT is pivotal in the preoperative diagnosis and perioperative staging of SBNETs. Surgical resection remains the gold standard for treatment. In special cases when coexisting with comorbidities such as scoliosis, an individualized treatment strategy should be made after being reviewed by a multidisciplinary discussion.

## 1. Introduction

Small bowel neuroendocrine tumors (SBNETs) represent the third largest subgroup among the gastrointestinal and pancreatic systems and are the most prevalent malignant neoplasms of the small intestine.^[1,2]^ Over the past 3 decades, the incidence has markedly increased, reaching 6.98 cases per 100,000 individuals.^[3]^ Multifocal occurrence is a notable characteristic of SBNETs, with approximately 25% of cases exhibiting this feature.^[4]^ Despite their indolent nature, many patients present with symptoms related to primary tumors, mesenteric invasion, and distant metastasis at the time of diagnosis.^[5]^ In this report, we present a unique case of SBNET associated with scoliosis and follow a literature review to elucidate the epidemiological features, clinical manifestations, imaging characteristics, pathological findings, and current treatment strategies for SBNETs, thereby giving some insights into diagnosis and treatment of such complex conditions.

## 2. Case presentation

### 2.1. History and clinical features

**Figure 1.**
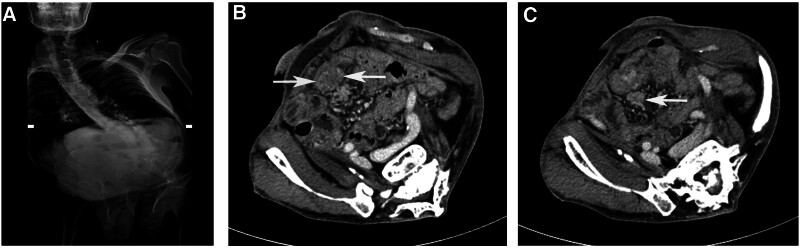
Images of spinal X-ray and abdominal CT. (A) Spinal X-ray indicates severe scoliosis. (B) Abdominal contrast-enhanced CT shows slight thickening and strengthening of the small intestine wall in the right low quadrant (arrow). (C) Abdominal CT shows a mesenteric mass near the primary disease (arrow).

A 69-year-old male patient presented to the gastroenterology department with a 10-month history of abdominal pain, nausea, vomiting, and weight loss. He had a previous medical history of poliomyelitis at the age of 6, resulting in severe scoliosis. He had been admitted to the other medical institutions multiple times due to recurrent abdominal pain and was diagnosed with small bowel obstruction over the past 10 months; however, the specific etiology remained unknown. Physical examination revealed significant abdominal deformity and tenderness in the epigastric region. Radiographic imaging demonstrated scoliosis on spine X-ray (Fig. 1A). Computed tomography scan of the abdomen revealed a slightly thickening and enhancement of the small bowel wall in the right lower quadrant (Fig. 1B) and a mesenteric mass (Fig. 1C). Screening tests for tumors and tuberculosis yielded negative results. Extensive diagnostic methods were employed including digestive tract radiography, gastroduodenoscopy, device-assisted enteroscopy, and colonoscopy; none showed evidence of neoplastic or inflammatory bowel disease was detected except for minor chronic inflammation observed in both stomach and colon.

### 2.2. Diagnosis and therapeutic process

**Figure 2.**
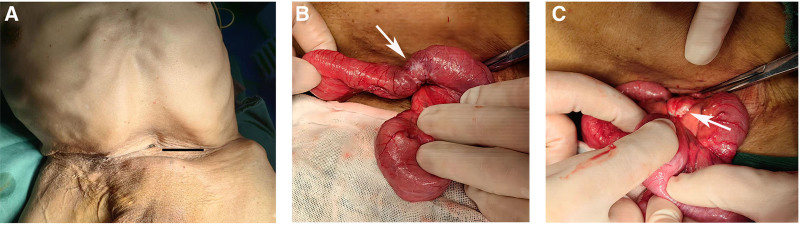
Intraoperative images. (A) Severe abdominal deformity caused by scoliosis, the surgical incision was selected in the left lower abdomen (marked line). (B) A small intestinal mass was found in the ileum (arrow) approximately 60 cm from the ileocecal junction. (C) A mesenteric mass and desmoplastic fibrosis can be seen near the small bowel lesion (arrow).

The most common cause of intestinal obstruction is adhesion formation following abdominal surgery. However, there is no documented history of surgical intervention in this patient’s medical records. Inflammatory bowel disease can also be a contributing factor to chronic small bowel obstruction, particularly affecting the terminal ileum. Nevertheless, no evidence of ileum ulcers or segmental small bowel thickening was observed in this patient. Based on the findings from computed tomography, it is suspected that the obstruction may be attributed to a neoplastic lesion within the small intestine. Performing a surgical procedure for this patient poses a major challenge due to the serious abdominal deformities caused by scoliosis. Decision-making was made according to the multidisciplinary team after weighted the clinical presentation and treatment goals, an exploratory laparotomy was conducted with an incision made in the left lower flank (Fig. 2A). A single tumor measuring 2 cm in diameter (Fig. 2B) and a mesenteric mass (Fig. 2C) was discovered in the ileum, located 60 cm from the ileocecal valve. Partial resection of the ileum and its mesentery was performed followed by reconstruction of the digestive tract through intestinal anastomosis.

### 2.3. Pathological characteristics

**Figure 3.**
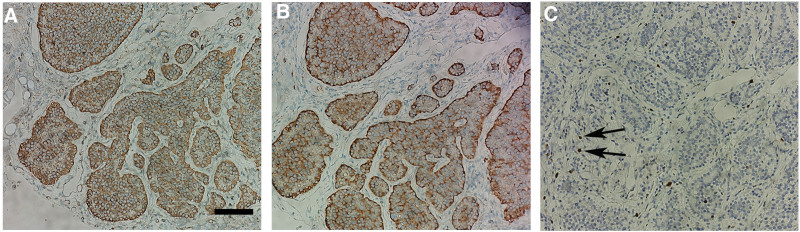
Pathological images. Immunohistochemical analysis of the tumor section demonstrated positive staining for CgA (A) and synaptophysin (B), and a Ki-67 (C, arrow) proliferation index ranging from 3% to 20%. Scale bar, 100 µm.

Immunohistochemical analysis of the tumor section demonstrated strongly positive staining for chromogranin A (CgA, Figure 3A), synaptophysin (Fig. 3B), and a Ki-67 proliferation index ranging from 3% to 20% (Fig. 3C), leading to the final diagnosis of a SBNET.

### 2.4. Follow-up

The patient experienced a smooth recovery without complications and was discharged 10 days after surgery. This patient did not received other therapy after surgery and no evidence of recurrence or metastasis at the 6 months of postoperative follow-up.

### 2.5. Discussion

SBNETs have a higher incidence than small intestinal adenocarcinomas, sarcomas, and lymphomas, making them the most prevalent malignant tumors of the small intestine.^[6]^ SBNETs are primarily classified based on their anatomical location, with the 2 main categories being those found in the foregut (duodenum) and those located in the midgut (jejunum and ileum). Due to the absence of a clear anatomical demarcation within the jejunum and ileum, precise classification within these regions remains further standardization. The literature indicates that the majority of SBNET cases originate in the terminal ileum, particularly within 100 cm of the ileocecal junction, comprising approximately 72% of reported cases.^[7]^ Several factors contribute to this phenomenon: (1) The unique intraluminal environment of the terminal ileum, where the ileocecal valve causes stasis and prolonged retention of intestinal contents, is also the primary site for the absorption of bile salts and vitamin B12. Additionally, substantial secretion of glucagon-like peptide-1 occurs here, with studies suggesting that glucagon-like peptide-1 plays a crucial role in the formation and maintenance of SBNETs.^[8]^ (2) Anatomical factors such as reduced villous architecture, the absence of circular folds, increased lymphoid follicles, and a higher prevalence of enterochromaffin cells may be associated with the pathogenesis of these tumors.^[9,10]^ SBNETs often present with nonspecific clinical symptoms in their early stages, and patients are typically diagnosed at an advanced stage, with 72% exhibiting regional invasion and 55% with distant metastases. Common clinical presentations include intestinal obstruction, mesenteric lymph node metastasis, ischemia due to mesenteric fibrosis, and distant metastases.^[11]^ Nonetheless, due to the slow growth and indolent nature of SBNETs, their prognosis is often more favorable compared to other malignant intestinal tumors.^[12]^ In the presented case, the patient experienced recurrent abdominal pain and intestinal obstruction. Preoperative CT scans and intraoperative exploration revealed a primary small intestinal tumor and a mesenteric mass located approximately 60 cm from the ileocecal junction, which aligns with commonly reported sites and clinical manifestations in the literature. Complicating the diagnosis and treatment planning, the patient had significant abdominal deformity caused by scoliosis, which presents substantial challenges for surgical intervention. Through a multidisciplinary collaborative approach, a comprehensive perioperative management strategy was devised, successfully leading to tumor resection and the patient’s subsequent recovery and discharge.

SBNETs are categorized based on their functional status, specifically into functional and nonfunctional types. Functional SBNETs can secrete serotonin, which is associated with symptoms such as diarrhea, flushing, bronchial asthma, and carcinoid heart disease.^[13,14]^ Nonspecific clinical manifestations may include abdominal pain, bloating, nausea, vomiting, weight loss, and gastrointestinal bleeding.^[15]^ Preoperative imaging, particularly triphasic contrast-enhanced multislice CT and MRI, plays a crucial role in the perioperative diagnosis and staging of SBNETs.^[16]^ CT imaging can identify primary tumors within the small intestine, characterized by intestinal wall thickening and masses. It can also reveal mesenteric masses and metastatic lymph nodes resulting from tumor infiltration, and assist in diagnosing distant metastases, making it vital for detecting multifocal lesions in the small intestine. Moreover, abdominal CT can delineate bowel obstructions caused by the primary tumor as well as mesenteric ischemia due to fibrosis in the mesentery.^[10,17–19]^ Endoscopic techniques, including electronic capsule endoscopy and double-balloon enteroscopy, are invaluable for diagnosing SBNETs and identifying multifocal disease.^[20]^ Additionally, 24-hour urinary 5-hydroxyindoleacetic acid levels and serum chromogranin A levels provide diagnostic value for SBNETs.^[17,21]^ Pathological diagnosis plays a crucial role in the identification of SBNETs. The main pathological markers include CgA, synaptophysin, and serotonin. According to the European Neuroendocrine Tumor Society guidelines, the Ki-67 proliferation index is essential for tumor grading and prognostication. Other available markers include somatostatin receptor-2, E-cadherin, p53, p27, and VEGF.^[10,22]^ In the case reported herein, the patient primarily exhibited symptoms of abdominal pain, bowel obstruction, and weight loss, without evidence of 5-hydroxytryptamine secretion indicative of the carcinoid syndrome, thus categorizing it as a nonfunctional type. Abdominal contrast-enhanced CT demonstrated a characteristic mesenteric mass, and immunohistochemical analysis of tumor tissue revealed positive staining for CgA and synaptophysin, with a Ki-67 proliferation index ranging from 3% to 20%, thereby classifying it as G2.

The gold standard treatment for SBNETs is surgical resection, which encompasses the excision of the primary small intestine tumor, mesenteric masses, and regional lymph nodes.^[10,23]^ Notably, over 80% of cases demonstrate mesenteric metastasis; thus, the extent of regional lymphadenectomy is a significant prognostic factor for SBNETs. Performing a comprehensive lymphadenectomy, including clearance of the lymph nodes at the roots of the superior mesenteric artery and superior mesenteric vein as well as those located posterior to the pancreas, has been shown to improve patient outcomes.^[24–27]^ Given that multifocal disease occurs in more than 40% of patients, meticulous preoperative imaging staging and thorough intraoperative exploration of the entire small intestine are of paramount importance.^[28,29]^ Despite approximately 60% of cases presenting with distant metastases at diagnosis, the 5-year survival rate for this subset remains at 57%. Furthermore, research suggests that surgical resection of the primary tumor in patients with metastatic SBNETs can extend survival.^[30]^ However, other research indicates that for stage IV cases without local tumor-related symptoms, localized surgery does not confer a survival benefit.^[31]^ Therefore, the formulation of treatment strategies for such cases necessitates collaboration among multidisciplinary expert teams. Other therapeutic interventions for SBNETs, such as somatostatin analogs, molecular targeted therapies, and peptide receptor radionuclide therapy, can delay disease progression.^[15]^ The European Neuroendocrine Tumor Society recommends that there is no indication for the use of specific adjunctive medications or neoadjuvant treatment methods in the postoperative management of patients with curative surgery.^[10]^ In the case presented, intraoperative exploration revealed a solitary tumor with mesenteric infiltration. Finally, excision of the primary tumor and mesenteric mass was performed alongside regional lymphadenectomy. The patient did not receive any specific pharmacological interventions following the surgery. No significant signs of recurrence have been observed after a 6-month follow-up.

## 3. Conclusion

SBNETs have a relatively low incidence and typically present with nonspecific clinical manifestations, primarily occurring in the distal ileum. Enhanced abdominal CT imaging plays a critical role in both diagnosis and preoperative staging, while small bowel endoscopy and laboratory molecular testing can provide diagnostic significance. Surgical intervention remains the first-line treatment for SBNETs. In cases classified as stage IV or accompanied by severe comorbidities such as scoliosis, developing a comprehensive treatment strategy through multidisciplinary collaboration is paramount.

## Author contributions

**Writing – original draft:** Cheng Deng.

**Writing – review & editing:** Jin Su.
